# Development of a marital self-disclosure programme for alleviating the fear of cancer recurrence in patients with gastric cancer and undergoing chemotherapy: a modified Delphi method

**DOI:** 10.3389/fpsyg.2024.1340915

**Published:** 2024-07-08

**Authors:** Ye Zhou, Chong Chin Che, Mei Chan Chong, Haiyan Zhao

**Affiliations:** ^1^Department of Nursing Science, Faculty of Medicine, Universiti Malaya, Kuala Lumpur, Malaysia; ^2^Nursing Department, Jingjiang People's Hospital, Taizhou, Jiangsu Province, China

**Keywords:** fear of cancer recurrence, gastric cancer, intervention, nursing, protocol, psychological, marital self-disclosure

## Abstract

**Aim:**

This study aimed to develop a marital self-disclosure programme to alleviate the fear of cancer recurrence in patients with gastric cancer who are undergoing chemotherapy.

**Design:**

Delphi method.

**Methods:**

Data from available literature and stakeholder interviews were utilised to formulate the initial draft of a marital self-disclosure programme aimed to alleviate the fear of cancer recurring in patients with gastric cancer and undergoing chemotherapy. A panel of experts subsequently conducted a two-round modified Delphi method to finalise the programme.

**Results:**

A total of 13 experts participated in the first round of consultation, while 11 experts were involved in the second round, as two experts withdrew due to unavailability. The response rates of both rounds of expert consultation were 100 and 84.62%, respectively, and the expert authority coefficients (Cr) of the programme were 0.83 and 0.84, respectively. The coordination coefficients of the expert opinions were 0.124 (χ^2^ = 61.214, *p* = 0.010) and 0.167 (χ^2^ = 69.668, *p* = 0.001) for each Delphi round. The average score of the second round was (4.545 ± 0.688) to (5.000 ± 0), with a full score ratio of 0.55–1.00. The coefficient of variation (CV) ranged from 0 to 0.031. Outcomes from both rounds of consultations were considered acceptable and credible. The finalised marital self-disclosure programme for alleviating the fear of cancer recurrence in patients with gastric cancer undergoing chemotherapy consists of two parts; disclosure guidance for patients and their spouse with nine items, and the structure and themes of marital self-disclosure with 31 items.

**Patient or public contribution:**

After two rounds of expert consultations, the marital self-disclosure programme for patients with gastric cancer undergoing chemotherapy is suggested to be scientifically valid and reliable. This programme is anticipated to potentially support patients and their spouses effectively by providing a reliable intervention plan focused on alleviating the fear of cancer recurrence.

## 1 Introduction

The advancement of postoperative chemotherapy for gastric cancer patients has increased the 5-year survival rate to 68% (Waters et al., 2014). Although the combination of surgery and chemotherapy has also saved patients’ lives, the high postoperative recurrence and metastatic rates have caused a great psychological burden, thus generating a negative psychological event (Waters et al., 2014), such as fear. The fear of a cancer reoccurring is defined as “fears and concerns in patients with cancer regarding a possible future recurrence of cancer and/or metastasis and/or progression” (Lebel et al., 2016). Simard et al. (2010) reported that about 72% of patients experienced different degrees of fear of cancer recurrence, of which about 46% were at the mild or moderate level, and about 7% had severe fears.

To the best of our knowledge, no psychological intervention programmes specifically studied the fear of cancer recurrence in Chinese patients with gastric cancer. To date, only one study conducted a cross-sectional survey investigating the fear of cancer recurrence in patients with gastric cancer, utilising a multiple linear regression analysis (Sicheng, 2020). The results demonstrated that the self-disclosure and intimacy of patients were influencing factors for their fear of cancer recurrence (Fan, 2021). Most programmes have focused on the impact of self-disclosure on the patient’s psychological aspect, ignoring the role of their spouse in the psychological state (Zhang et al., 2019; Rabin, 2020; Rafii et al., 2022), and thus, have failed to treat the patient and their spouse as a unit (Dandan et al., 2018). However, a spouse’s high level of fear of cancer recurrence not only affects the spouse’s own physical and mental health but deeply affects the patient’s health (Wang Dongyu et al., 2021).

Marital self-disclosure, also known as Open communication, refers to the ability of one partner to express his/her feelings or thoughts to the other person and to ask for responses for coping (Porter et al., 2009). Emotional disclosure is a central component of the marital emotional support that partners provide to each other (e.g., love, concern, and understanding; Porter et al., 2009). Married patients with cancer tend to regard their spouse as their most important confidant, and their marital intimacy is positively correlated with disease adaptation and psychological adjustment (Lepore and Revenson, 2007; Kershaw et al., 2008; Hagedoorn et al., 2011; Zhang, 2019). Discussing cancer-related issues as a couple may help partners better understand the patient’s needs and provide more effective support. Additionally, the partner may feel relief when the patient shares his/her concerns (Porter et al., 2019). Our research team conducted a systematic review, analysing 13 RCTs and found that although the structure and themes of marital self-disclosure are different, compared with cancer education and standard psychosocial care, psychosocial support and marital self-disclosure interventions had potential benefits on physical and psychological health, marital relationship, and self-disclosure ability. Additionally, compared to routine care, marital self-disclosure was shown to have certain benefits in alleviating the fear of cancer recurrence in breast cancer patients.

Therefore, it is critical to develop a feasible, effective, and reliable programme to alleviate the fear of cancer recurrence in Chinese patients with gastric cancer and their spouses to encourage remedial or preventive measures. Due to significant differences in race, cultural background, religious belief, and economic status between populations in the Western hemisphere versus China, it would be inaccurate to directly apply interventions for a married person with a Western cultural background to the Chinese population. The adaptability and accuracy of relevant theories, evaluation scales, and analysis of influencing factors must be carefully considered before concluding. Furthermore, research findings from other types of cancers cannot be generalised to patients with gastric cancer. Therefore, a tailored marital-based self-disclosure programme for Chinese patients with gastric cancer needs to be developed. Meanwhile, psychological intervention by psychiatrists or oncology mental health counsellors is costly, and resources are limited, making it difficult for every patient with psychological distress to receive prompt attention. Psychological nursing is one of the routine tasks of oncology nurses; they need to care for patients every day and spend a long time with them, making it easy to identify patients’ psychological distress (Lu, 2022). Nurses who have undergone psychological training can simultaneously possess knowledge of oncology and psychology, making them easily accessible resources for patients seeking psychological therapy. Therefore, it is necessary to establish a set of nurse-led psychological intervention methods tailored to oncology patients.

To achieve this, a literature analysis was first conducted to retrieve, screen, synthesise, and extract relevant content from existing literature. Interviews were additionally conducted with patients, their spouses, and nurses to develop a preliminary draft of a marital self-disclosure programme. Subsequently, utilising the Delphi method, experts in the fields of oncology, psychology, and gastroenterology were consulted to review and enhance the draft. Finally, the experts finalised the marital self-disclosure programme that aimed to alleviate the fear of cancer recurrence in patients with gastric cancer and undergoing chemotherapy.

## 2 Study design

The marital self-disclosure programme for patients with gastric cancer and their spouses was developed by analysing existing evidence, stakeholders’ interviews, and the Delphi method. During the first phase, the most up-to-date evidence on marital self-disclosure in patients with gastric cancer and their spouses was combined with outcomes from the stakeholder interviews to form the initial draft of the marital self-disclosure programme. In the second phase, the two-round Delphi method was employed to collect opinions from experts and refine items making up the content of the programme. This approach resulted in the development of a self-disclosure programme specifically designed to alleviate the fear of cancer recurrence in patients with gastric cancer and undergoing chemotherapy.

The Delphi method was utilised for consensus-building (Humphrey-Murto et al., 2017). It is an iterative process using several surveys in which researchers seek the consensus of experts by answering questions (McPherson et al., 2018). A two-round Delphi method was used to assess relevant experts across multiple regions in China, to establish a consensus for the marital self-disclosure programme that alleviated fear in patients with gastric cancer. This study complied with the Conducting and Reporting Delphi Studies (CREDES) standard (Jünger et al., 2017; Niederberger and Köberich, 2021).

**Figure 1:**
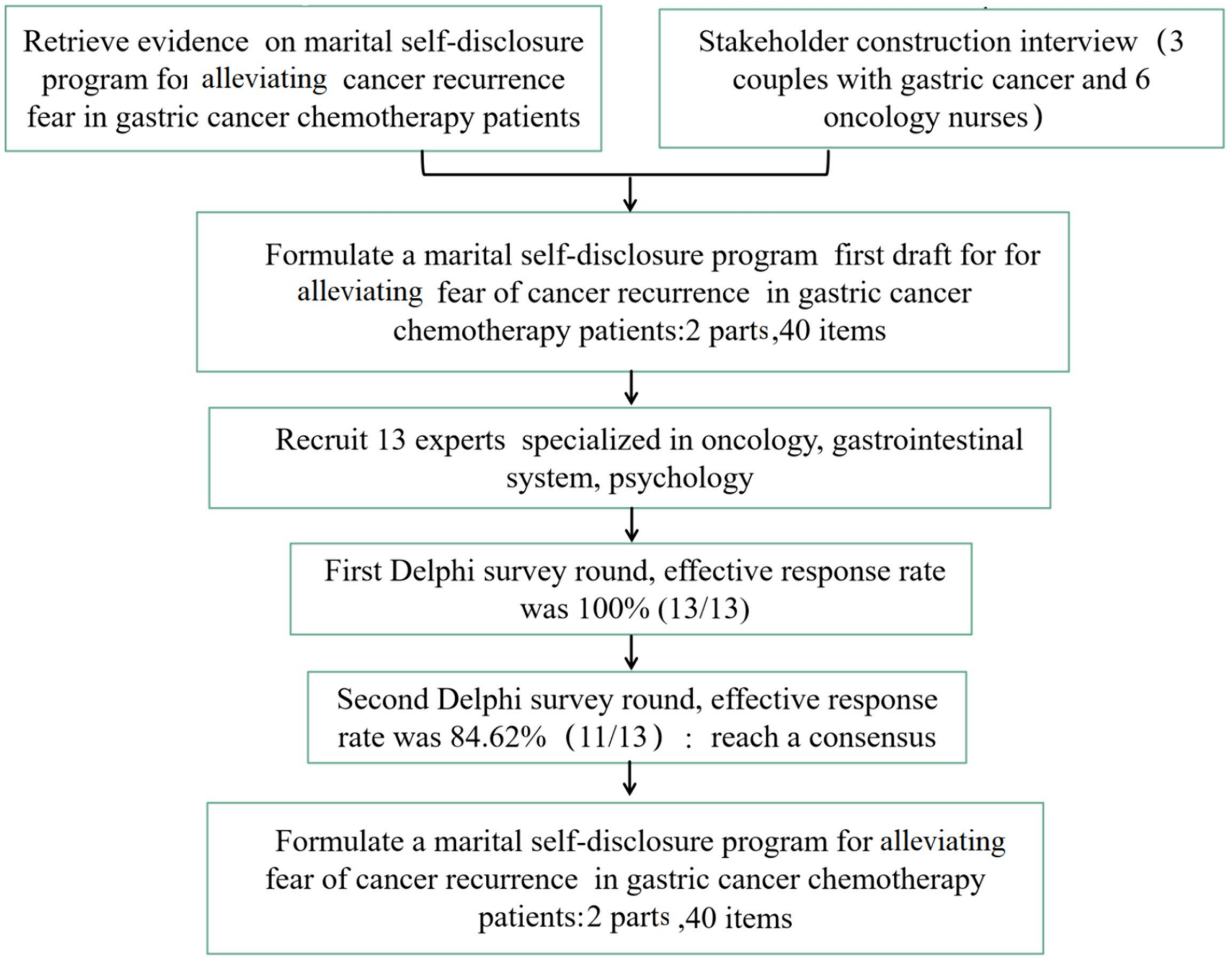
The research process for developing the marital self-disclosure programme.

The research team consists of six people, including two nurse managers, three nurses, and one researcher, of whom major in oncology, psychology, and the gastrointestinal system. The team members are mainly responsible for formulating research topics and programmes, compiling consultation structure and questionnaires for the experts, selecting the experts, and organising, summarising, and statistically analysing the consultation results. The research process for developing the marital self-disclosure programme is shown in Figure 1.

### 2.1 Literature search and analysis and stakeholder interview

Our team follows the Preferred Reporting Items for Systematic Reviews and Meta-Analysis (PRISMA) 2020 statement for Literature Search and Analysis (Page et al., 2020). The specific search strategy, retrieved literature, and analysis results can be found in the literature already published by our team (Zhou et al., 2023). Based on the literature analysis, we have initially developed a preliminary draft of marital self-disclosure’s framework, stages, timing, and disclosure process.

To gather data on MSD preference and suggestions, the researchers utilised purposive sampling to interview stakeholders, including patients with gastric cancer and their spouses and oncology nurses. The researcher conducted in-depth interviews with three patients with gastric cancer and their spouses to gain a deeper understanding of their experiences and MSD preference. Subsequently, the researcher conducted further interviews with six nurses from the oncology ward to gather their intervention preferences and suggestions of MSD. Interview questions mainly include: (1) Are the form, frequency, and timing of the MSD interventions, the evaluation time, and the themes in different intervention phases is appropriate? (2) What do you think should be noted during the process of MSD? (3) What topics do you think are beneficial for alleviating the FCR? (4) What additional aspects do you think need to be supplemented or addressed? Shown in the Supplementary Material A.

In terms of MSD intervention content, nursing staff believe that “it is necessary to fully respect the patient’s wishes, and in the process of intervention, attention should be paid to protecting the patient’s privacy.” “Pay attention to the expression, tone, and attitude; avoid sensitive vocabulary such as death; use gentle language, speak slowly, adopt a gentle tone, and maintain a sincere attitude. Establishing a trusting relationship with the patient is essential during the initial intervention.” “During the disclosure process, nurse should pay attention to listening and observe the emotional changes of couples. If conflicts or mutual accusations arise, communication should be stopped promptly.” Patients and their spouses believe that “mutual psychological guidance is crucial because emotions can mutually influence each other.” Regarding the frequency, Patients suggest that 2 to 6 sessions are more appropriate.

Regarding the timing of MSD intervention, patients emphasised that “the psychological pressure is greatest at the beginning of chemotherapy, and hope psychological intervention can be initiated as early as possible.” Nursing staff pointed out that “after receiving chemotherapy drugs, patients undergo prolonged intravenous infusion, which can make them feel uncomfortable, affecting their desire and mood for disclosure. Therefore, the appropriate timing for MSD intervention is when patients are hospitalised for chemotherapy but before receiving the drugs.” In line with the suggestions from the stakeholder interviews, the timing for MSD intervention sets as after hospitalisation but before the infusion of chemotherapy drugs.

Nursing staff believes “evaluation can be conducted after each intervention,” while patients find “the evaluation process a bit cumbersome. Despite their stable psychological feelings, the evaluation times are still in the early, middle, and late stages of intervention.” Both patients and nurses believe “a single form of psychological intervention may have a limited effect.” They suggest “incorporating additional methods such as homework assignments and WeChat reminders.” Based on the stakeholder interviews, besides verbal disclosure, written disclosure content has also been added. Additionally, during the patients’ home periods, WeChat reminders was included to encourage both patients and their spouses to engage in disclosure.

Based on the stakeholder interviews, more details were added to a preliminary draft of MSD, and then Delphi expert consultation was conducted.

### 2.2 Delphi expert consultation method

This study was conducted utilising the Delphi expert consultation approach from November 2022 to January 2023. The number of experts and survey rounds may vary according to the purpose of the study (McPherson et al., 2018), and a panel of 8 to 20 experts was generally recommended (Xu, 2016).

#### 2.2.1 Expert selection criteria

The programme’s content consisted of various fields, including oncology, psychology, and the gastrointestinal system. As research on marital self-disclosure is relatively new, especially intervention research in China, there are only a limited number of experts and research teams with significant theoretical and practical experiences in this area. Therefore, based on the availability of experts, 13 experts were recruited from eight tertiary A-level hospitals in four provinces and cities of Beijing, Jiangsu, Guangdong, and Yunnan.

The experts were selected based on the following criteria: (a) medical and nursing staff specialising in oncology, the gastrointestinal system, and psychology; (b) Experts have research or practical experience regarding dyadic coping (cancer patients and their spouses); (c) with a bachelor’s degree or higher; (d) intermediate or higher professional title in the medical or nursing position; (e) possessed extensive experience in the above fields and able to provide professional advice and guidance for this research; (f) had worked for more than ten years; and (g) willing to participate in this study. Before beginning consultations, the background and methods of this research were explained to the experts to obtain their support and cooperation.

#### 2.2.2 Expert consultation process

**Figure 2:**
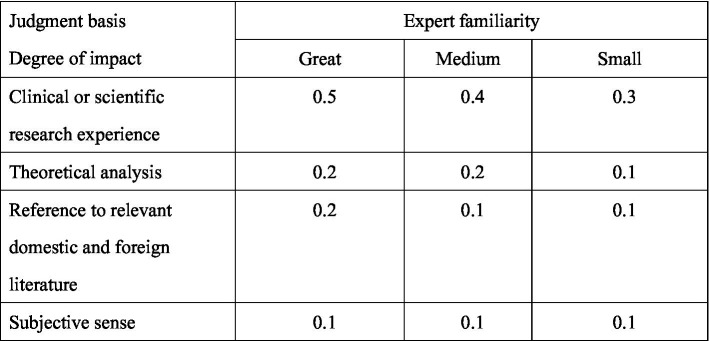
Expert familiarity and judgement basis table.

The expert’s consultation form consisted of four parts:

a.Instructions for the survey: Introduces the background, purpose, and content of the study, instructions for filling out the questionnaire, and the estimated reply time of the questionnaire.

b.Expert basic information questionnaire: A form for the experts to fill in their basic information, including name, professional title, phone number, gender, work institution, age, educational background, professional title, research field/professional direction, and working years.

c.Consultation form for opinions: The preliminary draft of the marital self-disclosure programme was provided for expert review. Experts were asked to rate the importance of each item using the 5-point Likert scale ranging from ‘1’ as extremely unimportant to ‘5’ as extremely important. A “Supplementary and Modified Comments” column was created for experts to provide their suggestions and opinions on the programme options.

d.Expert familiarity and judgement basis table: Experts were asked to rate their familiarity using very familiar (0.9), better familiar (0.7), moderately familiar (0.5), not very familiar (0.3), and very unfamiliar (0.1). The basis of judgement was clinical or scientific research experience (great-0.5, medium-0.4; small-0.3), theoretical analysis (great-0.2, medium-0.2; small-0.1), reference to relevant domestic and foreign literature (great-0.2, medium-0.2; small-0.1), subjective sense (great-0.1, medium-0.1; small-0.1). These consultation questionnaires were sent via e-mail and WeChat to the experts with two-week gaps between each round. The scoring rule of expert familiarity and judgement basis is shown in Figure 2 and Supplementary Material B.

After completion of the first round of expert consultation, results were collated, analysed, and discussed by the research team. The contents of the programme were adjusted and modified according to the feedback gathered. Next, materials for the second round of expert consultation were sent to the experts via e-mail and WeChat. The second-round consultation questionnaire included results of the previous round of expert consultation, opinions, and suggestions, with the changes to the MSD programme. However, there is no uniform definition for consensus (Hasson et al., 2000). A commonly used criterion is to set the consensus standard at no less than 75% agreement among group members or a score of at least 4 on a 5-point Likert scale (Chen et al., 2021).

### 2.3 Data analysis

The resulting data were analysed using the SPSS 26.0 software. Quantitative data are represented in percentage (%), and measurement data are represented by mean ± standard deviation (X ± S). Expert authority is expressed in the expert authority coefficient (Cr) and determined by the expert judgement basis coefficient (Ca) and expert familiarity coefficient (Cs), Cr = (Ca ± Cs)/2. The concentration degree of expert opinion is expressed by the arithmetic mean and full score ratio of items’ importance scores. The degree of coordination of expert opinions is expressed by standard deviation, coefficient of variation (CV), and Kendall’s *W* of each item importance score. *p* < 0.05 was considered statistically significant.

### 2.4 Ethical considerations

The study was approved by the Ethics Committee of Jingjiang People’s Hospital (KY 2022–167-01). The data collection instrument for the two-round Delphi method included a statement that described the purpose of the study and the completion of the questionnaire, which implied consent.

## 3 Research results

### 3.1 Results of literature analysis and stakeholder interviews

Our research team’s previous systematic review (Zhou et al., 2023) found that 13 randomised controlled trials (RCTs) indicated marital self-disclosure programmes were led by doctors and nurses who have undergone psychological training, as well as psychologists and other related professionals (Heinrichs et al., 2012; Zhang et al., 2019; Wang Dongyu et al., 2021; Duan et al., 2022). Cranstoun et al. (2024) suggested that oncology nurses who have received psychological training or are qualified as psychologists possess knowledge in both oncology and psychology, making them suitable candidates for implementing psychological oncology nursing. Therefore, the disclosure programme we designed is mainly led by nurses who have received psychological training and work in oncology. If psychological issues arise that cannot be resolved within the nurses’ scope of practice, timely referrals are made to psychiatric physicians. Data from this literature analysis further revealed that one-on-one, face-to-face communication, telephone communication, video conferencing, or mixed communications methods can be the intervention method (Porter et al., 2017; Badr et al., 2019; Manne et al., 2019). A scoping review from Cincidda et al. (2022) suggests that face-to-face psychological interventions for FCR are feasible, acceptable, and efficacious for managing FCR. However, there is no specific data on the interventions that are most effective when delivered remotely. Therefore, this study identifies one-on-one, face-to-face communication as the mode of marital self-disclosure.

The MSD intervention programme consisted of four brief sessions, each lasting 30 min, conducted over 3 to 4 months. The sessions allowed couples to discuss health-related issues, psychological/behavioural and social/interpersonal processes, their relationship as a couple, plans, and the affection they have for each other. Imagery and future-oriented exercises may also help remind couples of their coping ability and available resources, as well as facilitate discussions around difficult or sensitive topics (Gremore et al., 2021; Robieux and Bridou, 2022). Studies also indicated that since verbally disclosing certain matters to a partner may be challenging or problematic, a written disclosure would be more beneficial (Zhang et al., 2019; Yucel et al., 2021; Boyd et al., 2022). Additionally, prior to disclosure, patients with cancer and their spouses should be trained in marital communication skills that would help them express their thoughts and feelings about cancer better, as well as learn to accept and affirm each other’s feelings and opinions (Porter et al., 2017; Duan et al., 2022).

The stakeholder interviews conducted in this study revealed that one couple and one nurse preferred face-to-face interventions. Patients preferred to disclose information on the first day of hospitalisation and during their chemotherapy, with additional homework assigned during chemotherapy intervals. During disclosure, patients tended to express their experiences with the illness, feelings and thoughts, coping styles, positive thoughts and changes, and plans. Nurses suggested that reminding patients to disclose their information via phone calls or WeChat could be part of the MSD outlined.

### 3.2 Results of expert consultations

#### 3.2.1 General information of the experts

A total of 13 experts participated in the first round of expert consultation; 2 research experts’ research direction is psycho-oncology, and one of them has a psychological counselling certificate. Six of them are oncology nurses whose speciality or research direction is psycho-oncology. Three of them are full-time psychological nurses, as well as having rich experience in psychological nursing and psychological counselling, and two of them are psychological counsellors (one working in the outpatient clinic, and another working in the Gastroenterology Department), who have rich experience in psychological counselling of cancer patients (especially digestive tract tumours). During the second round of expert consultation, two experts (one is an oncology nurse whose speciality is psycho-oncology, and another is a psychological counsellor working in the outpatient clinic) withdrew due to supporting COVID-19 prevention and control, leaving a total of 11 participating experts. General information of the experts is shown in Table 1.

**Table 1:** General information of the experts.

		1st round (*n* = 13)	2nd round (*n* = 11)
Gender	Male	1	1
	Female	12	10
Age	30–39	5	4
	40–49	5	5
	≥50	3	2
Working Years	10~	7	6
	20~	3	3
	30~	3	2
Educational Background	Bachelor’s degree	5	4
	Master’s degree or above	8	7
Professional Title	Intermediate	1	1
	Deputy Senior	9	8
	Senior	3	2

#### 3.2.2 Response rate

In the first round, all 13 experts responded with valid questionnaires, resulting in a response rate of 100%. In the second round, 11 experts responded with valid questionnaires, resulting in a response rate of 84.62%.

#### 3.2.3 Authority coordination, concentrations and coefficients of variation

After two rounds of expert consultants, the degree of expert authority was high, at an authority coefficient (Cr) of 0.83 and 0.84, which was calculated based on a basis coefficient (Ca) of 0.94 and 0.95, and expert familiarity coefficient (Cs) of 0.72 and 0.72. The average concentration score of expert opinions in the second round was (4.545 ± 0.688)to (5.000 ± 0), which is considered high and had a full score ratio of 0.55–1.00. The coefficient of variation (CV) was 0 to 0.031.

The marital self-disclosure programme includes 2 parts: “disclosure guidance for patients and their spouse” and “the structure and themes of marital self-disclosure.” The average score of the importance of “disclosure guidance for patients and their spouse” was (4.636 ± 0.504)to (5.000 ± 0); with a full score ratio of 0.64–1.00; the CV was from 0 to 0.023. The average score of the importance of “the structure and themes of marital self-disclosure” was (4.545 ± 0.688) to (4.909 ± 0.302); with a full score ratio of 0.55–1.00, the CV was from 0.015 to 0.031. The CV of experts on the importance scores of each item ranged from 0.000 to 0.031, thus were all less than 0.25. The coordination coefficients of expert opinions for the MSD programme, “disclosure guidance for patient and their spouse” and “the structure and themes of marital self-disclosure” were 0.167 (χ^2^ = 69.668, *p* = 0.001), 0.210 (χ^2^ = 20.793, *p* = 0.014), and 0.150 (χ^2^ = 46.236, *p* = 0.016), respectively. The significance test of coordination coefficients of expert opinions is tabulated in Table 2, while concentrations and coefficients of variation of experts’ opinions in the second round are in Table 3.

**Table 2:** Significance test of coordination coefficients of expert opinions.

	Content	Items	Kendall’s W	χ^2^	*p*
1st round	Part 1: Disclosure guidance for patients and their spouse	10	0.181	21.159	0.012*
	Part 2: The structure and themes of marital self-disclosure	29	0.118	3.081	0.034*
	(overall) The marital self-disclosure programme	39	0.124	61.214	0.010*
2nd round	Part 1: Disclosure guidance for patients and their spouse	10	0.210	20.793	0.014*
	Part 2: The structure and themes of marital self-disclosure	29	0.150	46.236	0.016*
	(overall) The marital self-disclosure programme	39	0.167	69.668	0.001*

Significant, *p < 0.05.

**Table 3:** Concentrations and coefficients of variation of experts’ opinions in the second round.

Items	$\bar{X}$	S	CV
Disclosure guidance for patients and their spouse			
1 Disclosure guidance for the speaker	5.000	0.000	0.000
1.1 Share an experience associated with the fear of cancer recurrence that causes strong emotions	4.727	0.467	0.022
1.2 Sincerely expressing inner thoughts. The content of the expression may include feelings brought about by this experience, the reasons for these feelings, and the related needs.	5.000	0.000	0.000
1.3 Share the experience as detailed as possible, including the event itself and psychological feelings, and pay attention to sharing without subjective evaluation	5.000	0.000	0.000
1.4 Do not speak continuously; pause occasionally to allow your partner to respond with understanding and support.	4.818	0.405	0.019
2 Disclosure guidance for the listener	4.818	0.405	0.019
2.1 Try to stand in the position of the other party, understand their emotions during this experience, and accept and recognise the rationality of their emotions.	5.000	0.000	0.000
2.2 Avoid immediately solving problems or giving suggestions. Focus on the patient’s feelings, pay attention to listening and expressing empathy, and reveal own thoughts when necessary to encourage the other party to continue talking.	4.818	0.405	0.019
2.3 Use reflective listening and summarise what the speaker has said instead of offering comforting or problem-solving advice.	4.636	0.505	0.023
2.4 Pay attention to your tone of speaking, maintain eye contact, and nod to express understanding.	5.000	0.000	0.000
The structure and themes of marital self-disclosure			
1 The structure of marital self-disclosure	4.909	0.302	0.015
1.1 Verbal disclosure (couple, face-to-face)	4.818	0.405	0.019
1.2 Written disclosure (patient)	4.545	0.688	0.031
2 Frequency of marital self-disclosure	4.545	0.522	0.024
2.1 When the patient is hospitalised for chemotherapy, the nurse-led self-disclosure frequency for the couple is once a month (4 cycles in total). In addition to the fixed frequency, the couple can express their emotions at any time they need to.	4.727	0.467	0.022
2.2 During the intermittent period of chemotherapy at home, the frequency of marital self-disclosure between the patient and their spouse is once a week.	4.727	0.467	0.022
2.3 Verbal self-disclosure lasts for 20 to 30 min each time.	4.545	0.688	0.031
2.4 Written self-disclosure lasts for 20 to 30 min each time.	4.636	0.505	0.023
3 The themes of marital self-disclosure	4.636	0.505	0.023
3.1Personal Emotional Expression	4.909	0.302	0.015
3.1.1 The patients and their spouse express their thoughts and feelings about the patient’s illness	4.909	0.302	0.015
3.1.2 couple expresses concerns about cancer recurrence or progression	4.818	0.405	0.019
3.1.3 Couples express other emotions	4.909	0.302	0.015
3.1.4 Your inner thoughts and feelings during the illness (can be written in terms of inner emotions, relationships with others, personal sentiments, concerns about the fear of cancer recurrence, etc.)	4.909	0.302	0.015
3.2 Social Cognition Expression	4.545	0.522	0.024
3.2.1 Couples reveal the impact of cancer on the family or social functions	4.818	0.405	0.019
3.2.2 Disclose specific concerns about the fear of cancer recurrence around social functioning	4.636	0.505	0.023
3.2.3 The impact of fear of cancer recurrence on the family life and social life of oneself and family members	4.909	0.302	0.015
3.2.4 Write your specific worries and concerns about the fear of cancer recurrence and its impact on you (e.g., negative emotions, impact on yourself and your family, etc.)	4.818	0.405	0.019
3.3 Benefit Discovery	4.909	0.302	0.015
3.3.1 The couple reveals any specific concerns about the progression of the disease	4.727	0.647	0.031
3.3.2 couple reveals mutual benefits from the illness experience	4.909	0.302	0.015
3.3.3 Couples reveal positive changes that occurred during treatment	4.909	0.302	0.015
3.3.4 Write the positive changes you have experienced from your illness (e.g., your emotions, relationships with others, treatment feelings, etc.)	4.909	0.302	0.015
3.4 Outlook to The Future	4.727	0.467	0.022
3.4.1 couple reveals changing views on cancer recurrence	4.818	0.405	0.019
3.4.2 Patients and their spouse disclose their future family plans	4.727	0.467	0.022
3.4.3 Patients and their spouses express their needs and formulate a plan to manage negative emotions and change unhealthy lifestyle	4.818	0.405	0.019
3.4.4 Please summarise and write your emotional changes during the treatment of the disease and your hopes for the future (e.g., emotional changes of disease recurrence or progression, personal hopes, etc.).	4.818	0.405	0.019

Significant, *p < 0.05.

#### 3.2.4 Modification of the MSD programme after expert consultations

After research team discussion, the Modification of the MSD programme after Expert Consultations shown in the Table 4.

**Table 4:** Modification of the marital self-disclosure programme after expert consultations.

Original description	Experts opinions	Modification
The first round		
“1 speaker disclosure requirements” “2 listener disclosure requirements”	Does not conform to the Chinese description of habit	1 Disclosure guidance for the speaker 2 Disclosure guidance for the listener
“1.1 Share a subject-related experience that causes strong emotions”	It is suggested that the term “subject” be described more clearly	“1.1 Share an experience associated with the fear of cancer recurrence that causes strong emotions”
“1.2 Sincere expression of the true thoughts”	It is recommended that the content of the expression be related to the illness and that the expression have a specific direction.	“1.2 Sincerely expressing inner thoughts. The content of the expression may include feelings brought about by this experience, the reasons for these feelings, and the related needs.”
“3.2.3 Patients and their spouse disclosure the impact of fear of recurrence on both lives.” “3.4.2 Patients and their spouse disclosure their future plans.”	It is recommended that couples’ disclosure regarding the impact on their lives and their future plans should be centred around the family.	“3.2.3 The impact of fear of cancer recurrence on the family life and social life of oneself and family members” “3.4.2 Patients and their spouse disclose their future family plans”
“2.1 Try to stand in the other person’s shoes and understand the other person’s experience.”	Experts suggested that it is not about “understanding the other person’s experience,” but rather about understanding “the emotions and feelings of the other person during that experience, and accepting and recognising the legitimacy of those emotions.”	“2.1 Try to stand in the position of the other party, understand their emotions during this experience, and accept and recognise the rationality of their emotions.”
The second round		
“2.1 The frequency of nurse-led couple self-disclosure during inpatient chemotherapy is once per month (in 4 cycles)”	In addition to the fixed frequency, emotional disclosure can be conducted on an as-needed basis when either spouse feels the need.	“2.1 When the patient is hospitalised for chemotherapy, the nurse-led self-disclosure frequency for the couple is once a month (4 cycles in total). In addition to the fixed frequency, the couple can express their emotions at any time they need to.”

The results of the second round of Delphi consultation revealed that the average scores for all indicators were no less than 4. Therefore, it is considered that a consensus was reached among the experts, and a third round of Delphi consultation was not conducted.

### 3.3 Content of marital self-disclosure programme for alleviating the fear of cancer recurrence in patients with gastric cancer undergoing chemotherapy

The research team conducted group discussions and formulated the final plan for the disclosure programme based on the results from the Delphi method of expert consultation. MSD was conducted in a quiet and cozy rest room with one-on-one interactions, beginning with verbal disclosure, followed by written disclosure. Patients written their experiences based on the theme of written disclosure. Verbal self-disclosure lasts for 20 to 30 min each time, and written self-disclosure lasts for 20 to 30 min each time. When the patient is hospitalised for chemotherapy, the frequency of nurse-led MSD is once a month (4 cycles in total). In addition to the fixed frequency, the couple can disclosure their emotions whenever needed.

During the intermittent period of chemotherapy, the frequency of MSD between the patient and their spouse at home is once a week. The content of MSD for patients and their spouses includes guidance on specific methods for disclosure (refer to Table 5) and the structure of MSD. Four cycles of MSD are based on four themes: Personal Emotional Expression, Social Cognition Expression, Benefit Discovery, Outlook to The Future. According to each stage’s verbal disclosure (couple) goals, the corresponding disclosure outlines are formulated (refer to Table 6).

**Table 5:** Disclosure guidance for patients and their spouses.

Disclosure guidance for the speaker	Share an experience associated with the fear of cancer recurrence that causes strong emotions Sincerely expressing inner thoughts. The content of the expression may include feelings brought about by this experience, the reasons for these feelings, and the related needs. Share the experience as detailed as possible, including the event itself and psychological feelings, and pay attention to sharing without subjective evaluation. Do not speak continuously; pause occasionally to allow your partner to respond with understanding and support.
Disclosure guidance for the listener	Try to stand in the position of the other party, understand their emotions during this experience, and accept and recognise the rationality of their emotions. Avoid immediately solving problems or giving suggestions. Focus on the patient’s feelings, pay attention to listening and expressing empathy, and reveal own thoughts when necessary to encourage the other party to continue talking. Use reflective listening and summarise what the speaker has said instead of offering comforting or problem-solving advice. Pay attention to your tone of speaking, maintain eye contact, and nod to express understanding.

Both the patient and their spouse take turns to be the speaker and listener.

**Table 6:** The structure and themes of marital self-disclosure.

Phase 1 (first chemotherapy)	
Theme	Personal emotional expression
Verbal disclosure (couple) goals	1. The patients and their spouse express their thoughts and feelings about the patient’s illness 2. Couples express concern about cancer recurrence or progression 3. Couples express other emotions
Disclosure outlines	a.How have you felt since (your partner) became ill? b.Are you worried about the progression of the disease? What are the specific worries? c.What other emotions did you have during the treatment?
Written disclosure (patient)	Your inner thoughts and feelings during the illness (for example, inner emotions, relationships with others, personal sentiments, concerns about the fear of cancer recurrence, etc.)
Phase 2 (second chemotherapy)	
Theme	Social cognition expression
Verbal disclosure (couple) goals	4. Couples reveal the impact of cancer on family or social functions 5. Disclose specific concerns about the fear of cancer recurrence around social functioning 6. The impact of fear of cancer recurrence on family life and social life of oneself and the family members
Disclosure outlines	a. What impact does the (your partner’s) illness have on your family, work, and social interactions? b. Are you worried about the progression of the disease? What are the specific worries? c. What other emotions did you have while undergoing treatment?
Written disclosure (patient)	Please write down your specific worries and concerns about the fear of cancer recurrence, and its impact on you (for example, negative emotions, impact on yourself and your family, etc.)
Phase 3 (third chemotherapy)	
Theme	Benefit discovery
Verbal disclosure (couple) goals	7. Couples reveal any specific concerns about the progression of the disease 8. Couples reveal mutual benefits from experiencing the illness 9. Couples reveal positive changes that occurred during treatment
Disclosure outlines	a. Did you benefit from the experience of being sick? What are the specific benefits? What were the good influences on you? b. What positive emotions did you have after (your partner) got sick? c. What positive changes have you made during your (partner’s) treatment?
Written disclosure (patient)	Write down the positive changes you have experienced from your illness (you can write about your emotions, relationships with others, treatment feelings, etc.)
Phase 4 (fourth chemotherapy)	
Theme	Outlook to The Future
Verbal disclosure (couple) goals	10. Couples reveal changing views on cancer recurrence 11. Patients and their spouse disclose their future family plans 12. Patients and their spouses express their needs and formulate a plan to manage negative emotions and change unhealthy lifestyles.
Disclosure outlines	a. What are your plans for the future? b. What other needs do you have for alleviating disease recurrence and what are your plans to manage negative emotions and change unhealthy lifestyles? c. What other emotions did you have during the treatment?
Written disclosure (patient)	Summarise and write down your emotional changes during the treatment of the disease and your hopes for the future (you can write emotional changes related to disease recurrence or progression, personal hopes, etc.).

## 4 Discussion

Thirteen experts were included in a consultation process utilising the Delphi method. These experts came from eight tertiary A-level hospitals in four provinces in China, with a wide geographical distribution. Two research experts specialise in psycho-oncology, while six are oncology nurses whose professional or research focus is in psycho-oncology. Among them, three are full-time psycho-oncology nurses with extensive experience in psychological care and counselling. Two are psychological counsellors (one works in outpatient services, the other in gastroenterology), both with rich experience in psychological counselling, especially for gastrointestinal cancer patients. The work content or research focus of these experts is suitable for consultation in this study. Of the 13 experts involved, 61.54% (8/13) hold a master’s degree or above, while 92.31% (12/13) hold senior titles. Despite having an intermediate professional title, one oncology nurse expert has 10 years of work experience and a broad research scope related to this study. Thus, she was also included in the expert consultation team.

Outcomes from the expert consultants demonstrated that the familiarity coefficient, judgement basis and authority coefficients of the programme were rated as good. This indicated that the experts were very familiar with the research content and possessed extensive experience in both theoretical and practical aspects of this field. Moreover, the experts scored the importance of each item at a high level, while their opinions were well-coordinated and consistent, suggesting that the opinions were credible and desirable.

Enhancing the dyadic coping ability of gastric cancer patients and alleviating their fear of cancer recurrence is crucial to motivate remedial actions. To date, there are limited psychological interventions that appear to be effective for alleviating the fear of cancer recurrence in patients with gastric cancer and their spouse. This is the first and only intervention programme with the explicit aim of improving the fear of cancer recurrence in Chinese patients with gastric cancer. The marital self-disclosure intervention programme focuses on personal emotional expression, social cognition expression, benefit discovery, and outlook on the future among survivors, patients, and their spouses.

Several advantages are present in the developed programme and are expected to contribute to its positive impact on alleviating the fear of cancer recurrence among the specific group of patients studied. As the programme was developed in collaboration with Chinese medical staff, and patients, it is culturally appropriate and relevant. This stakeholder input has enabled the programme to address the unique needs and concerns of patients with gastric cancer and undergoing chemotherapy in the Chinese context. According to Sicheng (2020), patients with gastric cancer in the first, third, and sixth stages of chemotherapy feared less of a cancer recurrence with more family support. Therefore, the current programme spans four chemotherapy cycles to encompass the early to late chemotherapy sessions. Furthermore, the programme involves providing communication training to patients and their spouses on self-disclosure. By mastering the methods of self-disclosure while married, couples can engage formally and effectively apply these techniques to everyday communication (Gremore et al., 2021).

The themes included in the self-disclosure programme were established appropriately and reasonably, considering patient willingness and information from relevant literature. This approach helps ensure that the themes are relevant to the needs and preferences of patients with gastric cancer and supported by existing research (Zhang et al., 2019; Wang Dongyu et al., 2021). Previous studies found that patients with cancer exhibited self-disclosure avoidance (Northouse et al., 2000) as well as social isolation (Ettridge et al., 2018). Therefore, the first and second self-disclosure themes were set around “Personal Emotional Expression” and “Social Cognition Expression.”

As positive psychology develops and research deepens, people have realised that negative life events, such as cancer, can also bring about certain positive changes for patients (Stanton and Bower, 2015), such as cultivating healthy lifestyles, establishing new social relationships, and increasing disease knowledge. Wang et al. (2015) refer to these positive changes brought about by illness as “benefit finding” (BF). Planning positively for the future and guiding couples to adopt a positive outlook on cancer recurrence can encourage them to adjust negative emotions, change unhealthy habits, and plan for future family and social integration. Therefore, this study takes “Benefit Discovery” and “Outlook to The Future” as disclosure themes, aiming to alleviate the psychological trauma and stress caused by illness, encourage patients to face the disease with optimism, adopt effective behaviours to cope with the disease and contribute to physical and mental recovery. This has significant implications for reducing the fear of cancer recurrence (Stein et al., 2014; Cavell et al., 2016). Therefore, in addition to emotional disclosure, intervention themes also included “Benefit Discovery” and “Outlook to The Future. “The programme also combines both verbal and written self-disclosure to address the needs of patients who may struggle to verbally express or partially conceal their emotions. By using written self-disclosure, patients are encouraged to fully and accurately express their fears and concerns about cancer recurrence (Zhang et al., 2019; Yucel et al., 2021; Boyd et al., 2022).

Finally, this marital self-disclosure intervention programme was specifically designed for implementation by nurses, possibly appealing to medical staff seeking greater flexibility and inclusivity in their work. This intervention equips nurses with the necessary skills to teach patients and their spouses to apply theories of respect, empathy, and positive attention in emotionally driven communication and marital interactions. With this approach, nurses can also establish stable, friendly, and trusting relationships between themselves as caregivers and patients.

## 5 Limitations

Several limitations have been identified in the current study. Firstly, it is important to note that the field of marital self-disclosure intervention is relatively new, with only a limited number of scholars specialising in this area, including the 13 experts included in the study. However, the selected experts have extensive and relevant experience spanning over 10 years and a strong research background. As a result, the content of the current marital self-disclosure programme can be regarded as highly authoritative and informed by a wealth of expertise.

Secondly, the marital self-disclosure programme for couples was only developed using the Delphi method and has not yet been tested on patients with gastric cancer and their spouses. Therefore, its effectiveness in this specific context is currently unknown. To address this gap, further research utilising a randomised controlled trial (RCT) research is necessary to rigorously evaluate the programme’s effectiveness.

Lastly, this research represents a long-term programme and, thus, requires a few practical considerations to be addressed. For instance, ensuring patient compliance during the implementation process and integration into regular nursing work without compromising efficiency. Moreover, all experts involved in this Delphi method study were of Chinese ethnicity, limiting the programme’s applicability and generalizability outside China.

## 6 Conclusion

In this study, the preliminary draft of the marital self-disclosure programme was developed through literature analysis and stakeholder interviews. The final programme was developed after two rounds of expert consultation, and the results were credible and reliable. The marital self-disclosure programme can be potentially applied to patients with gastric cancer and their spouses, to evaluate its impact on alleviating the fear of cancer recurrence. To further validate the programme’s effectiveness, feasibility, and generalizability, a health economics evaluation could be conducted. The evaluation would provide valuable insights into the costs and benefits associated with the programme, and help determine whether it is a viable intervention for a broader patient population.

## Data availability statement

The original contributions presented in the study are included in the article/Supplementary material, further inquiries can be directed to the corresponding author.

## Ethics statement

The studies involving humans were approved by the Ethics Committee of Jingjiang People’s Hospital. The studies were conducted in accordance with the local legislation and institutional requirements. The participants provided their written informed consent to participate in this study.

## Author contributions

YZ: Writing – original draft, Methodology, Investigation, Formal analysis, Data curation, Conceptualization. CC: Writing – review & editing, Supervision. MC: Writing – review & editing, Supervision. HZ: Writing – review & editing, Methodology, Investigation, Funding acquisition, Formal analysis, Conceptualization.
