# Supplementary material for: Development of a marital self-disclosure programme for alleviating the fear of cancer recurrence in patients with gastric cancer and undergoing chemotherapy: a modified Delphi method
Source: Front Psychol. 2024 Jul 8;15:1340915. doi: 10.3389/fpsyg.2024.1340915 (PMC11262412; doi:10.3389/fpsyg.2024.1340915)
Supplement: Supplementary file 1 [file Data_Sheet_1.PDF]

Supplementary Material A

Stakeholder Interview Questions

**1. Intervention Forms**<br>- Are the forms of intervention, such as verbal disclosure and written disclosure, appropriate?
**2. Intervention Frequency and Timing**<br>- 2.1 How many times do you think the intervention program should be implemented? Should there be any additions or deletions?<br>- 2.2 At what points in time do you think it is feasible to conduct MSD ?
**3. Evaluation Timing**<br>- When do you think it is feasible to evaluate the effectiveness of the intervention program during its implementation?
**4. Other Considerations**<br>- 4.1 Are there any other aspects of the intervention program that you think need to be supplemented or taken into consideration?<br>- 4.2 What should be noted during the process of mutual disclosure between spouses?<br>- 4.3 What topics do you think are beneficial to discuss in alleviating the fear of cancer recurrence?

Supplementary Material B

First Round Delphi Expert Consultation Questionnaire

Dear Expert,

Hello, I am the Head Nurse of the Internal Medicine Department at Jingjiang People's Hospital. I am currently conducting a study titled "The Impact of Couples' Self-Disclosure Based on the Dual Coping Theory on Fear of Cancer Recurrence in Gastric Cancer Patients." The first part of this study involves developing a couples' self-disclosure plan for gastric cancer patients and their spouses, specifically targeting the fear of cancer recurrence.

In 2020, there were 1,089,103 new cases of gastric cancer worldwide, with 768,793 deaths, accounting for 5.6% of the total incidence rate and 7.7% of the total number of deaths. This poses a serious threat to public health. The postoperative recurrence and metastasis rates can be seen as negative life events that cause psychological impacts on patients and their spouses, such as anxiety, depression, avoidance, and self-perceived burden. Fear of cancer recurrence is the most common psychological response among cancer patients, referring to a psychological state where patients are afraid, worried, or anxious about cancer recurrence or progression. It is subjective, non-specific, and multidimensional, and can occur during the onset, development, and recovery stages of the disease. It is also one of the primary psychological needs for which cancer patients currently lack supportive care. More than 48% of gastric cancer patients experience mild to moderate fear of cancer recurrence, while about 7% experience severe fear. A meta-analysis published in 2021 confirmed that reasonably controlling the medical behavior related to fear of cancer recurrence will bring significant cost benefits. Marital emotional disclosure involves expressing personal, private, and emotional information to one's spouse, forming the basis of emotional support between couples. It is a core component of the emotional support (such as love, care, and understanding) that partners provide to each other. For married or intimately involved cancer patients, the relationship with their partner plays a crucial

role in adapting to the disease and alleviating the stress of the illness. Therefore, it is essential to develop an appropriate and targeted couples' self-disclosure plan.

Through literature review, semi-structured interviews, and other methods, I have preliminarily drafted a couples' self-disclosure plan, including 42 items. Knowing of your esteemed academic accomplishments and reputation in this field, I sincerely invite you to be a consulting expert for this study. Your suggestions and opinions are vital to this research.

The plan is divided into three parts. The first part is the guidance method for couples' self-disclosure for patients and their spouses. The second part covers the forms and themes of couples' self-disclosure. You are invited to rate the importance of each dimension and item in the plan. The third part is the expert's basic information and self-evaluation form for expert authority. All your personal information will be used solely for research purposes. Please fill out the following form according to the instructions. Kindly provide your feedback via email within 10 days of receiving the plan.

Thank you sincerely for your support and assistance. I wish you success in your work and happiness in life!

Yours sincerely,

Researcher: Zhao Haiyan

Nursing Department, Jingjiang People's Hospital

Instructions for Filling Out the Form: Each dimension and item is rated on a scale

of five levels of importance: Very Important (5 points), Important (4 points), Neutral (3 points), Unimportant (2 points), Very Unimportant (1 point). Please mark the corresponding box according to your opinion. If you find any dimension or item content inappropriate or improperly expressed, please provide your suggestions for modification in the corresponding modification column. If there are any items that need to be added, please supplement them in the remarks section below.

Table 1：Disclosure guidance for patients and their spouse

Items	Importance Rating					Expert Review Comments
	Very Important	Import ant	Modera te	Unimpo rtant	Very Unimport ant	
1 Speaker disclosure requirements	5	4	3	2	1	
1.1 Share a subject-related experience that causes strong emotions	5	4	3	2	1	
1.2 Sincere expression of the true thoughts	5	4	3	2	1	
1.3 Share the experience as detailed as possible, including the event itself and psychological feelings, and pay attention to sharing without subjective evaluation	5	4	3	2	1	
1.4 Do not speak continuously; pause occasionally to allow your partner to respond with understanding and support.	5	4	3	2	1	
2 Listener disclosure requirements”	5	4	3	2	1	

2.1 Try to stand in the other person's shoes and understand the other person's experience.	5	4	3	2	1	
2.2 Avoid immediately solving problems or giving suggestions. Focus on the patient's feelings, pay attention to listening and expressing empathy, and reveal own thoughts when necessary to encourage the other party to continue talking.	5	4	3	2	1	
2.3 Use reflective listening, summarize what the speaker has said instead of offering comforting or problem-solving advice.	5	4	3	2	1	
2.4 Pay attention to your tone of speaking, maintain eye contact, and nod to express understanding.	5	4	3	2	1	
**Add/Delete entry:**						

Table 2：The structure and themes of marital self-disclosure

Items	Importance Rating					Expert Review Comments
	Very Important	Important	Moderate	Unimportant	Very Unimportant	
1 The structure of marital self-disclosure	5	4	3	2	1	
1.1 Verbal disclosure (couple, face-to-face)	5	4	3	2	1	
1.2 Written disclosure (patient)	5	4	3	2	1	
3 The themes of marital self-disclosure	5	4	3	2	1	
2.1Personal Emotional Expression	5	4	3	2	1	
2.2Social Cognition Expression	5	4	3	2	1	
2.3Benefit Discovery	5	4	3	2	1	
2.4Outlook to The Future	5	4	3	2	1	
2 Frequency of marital self-disclosure	5	4	3	2	1	
2.1 The frequency of nurse-led couple self-disclosure during inpatient chemotherapy is once per month (in 4	5	4	3	2	1	

cycles)						
2.2 During the intermittent period of chemotherapy at home, the frequency of marital self-disclosure between the patient and their spouse is once a week.	5	4	3	2	1	
2.3 Verbal self-disclosure lasts for 20 to 30 minutes each time.	5	4	3	2	1	
2.4 Written self-disclosure lasts for 20 to 30 minutes each time.	5	4	3	2	1	
**Add/Delete entry：**						
**First self-disclosure (Personal Emotional Expression)**<br>**Verbal self-disclosure goal**						
3.1.1 The patients and their spouse express their thoughts and feelings about the patient's illness	5	4	3	2	1	
3.1.2 Couple expresses concerns about	5	4	3	2	1	

cancer recurrence or progression						
3.1.3 Couples express other emotions	5	4	3	2	1	
**Written self-disclosure（patient）**						
Your inner thoughts and feelings during the illness (can be written in terms of inner emotions, relationships with others, personal sentiments, concerns about the fear of cancer recurrence, etc.)	5	4	3	2	1	
**Add/Delete entry：**						
**Second self-disclosure（Social Cognition Expression）：**<br>**Verbal disclosure goal**						
3.2.1 Couples reveal the impact of cancer on the family or social functions	5	4	3	2	1	
3.2.2 Disclose specific concerns about	5	4	3	2	1	

the fear of cancer recurrence around social functioning						
3.2.3 Patients and their spouse disclosure the impact of fear of recurrence on both lives.	5	4	3	2	1	
**Written self-disclosure（patient）**						
Write your specific worries and concerns about the fear of cancer recurrence, and its impact on you (fe.g. negative emotions, impact on yourself and your family, etc.)	5	4	3	2	1	
**Add/Delete entry：**						
**Third self-disclosure（Benefit Discovery）**<br>**Verbal disclosure goal**						
3.3.1 The couple reveals any specific	5	4	3	2	1	

concerns about the progression of the disease						
3.3.2 Couple reveals mutual benefits from the illness experience	5	4	3	2	1	
3.3.3 Couples reveal positive changes that occurred during treatment	5	4	3	2	1	
**Written self-disclosure（patient）**						
Write the positive changes have experienced from your illness (e.g. your emotions, relationships with others, treatment feelings, etc.)	5	4	3	2	1	
**Add/Delete entry：**						
**Fourth self-disclosure（Outlook to The Future）**<br>**Verbal disclosure goal**						
3.4.1 Couple reveals changing views on	5	4	3	2	1	

cancer recurrence						
3.4.2 Patients and their spouse disclosure their future plans.	5	4	3	2	1	
3.4.3 Patients and their spouses express their needs and formulate a plan to manage negative emotions and change unhealthy lifestyle	5	4	3	2	1	
**Written self-disclosure（patient）**						
Please summarize and write your emotional changes during the treatment of the disease and your hopes for the future (e,g, emotional changes of disease recurrence or progression, personal hopes, etc.).	5	4	3	2	1	
**Add/Delete entry：**						

Table 3: Expert Basic Information Consultation Form

**Name**	**Position**	**Contact Number**
**Gender**	**Email**	**Work address**
**Age**	□30-39 years □40-49 years □≥50 years	
**Education**	□Bachelor's Degree □Master's Degree or Higher	
**Professional Title**	□Intermediate □Associate Senior □Senior	
**Research Field/Professional Direction**		
**Years of work Experience**	□10-19 years □20-29 years □≥30 years	

Table 4: Please select your familiarity with the study and mark with "✓" in the corresponding box

**Familiarity Level**	**Very Familiar (0.9)**	**Quite Familiar (0.7)**	**General Familiar (0.5)**	**Not Very Familiar (0.3)**	**Not Familiar (0.1)**
Expert Self-Evaluation					

Table：Figure 2. Expert familiarity and judgment basis table

**Judgment basis**	**Expert familiarity**		
**Degree of impact**	Great	Medium	Small
**Clinical or scientific research experience**	0.5	0.4	0.3
**Theoretical analysis**	0.2	0.2	0.1

**Reference to relevant domestic and foreign literature**	0.2	0.1	0.1
**Subjective sense**	0.1	0.1	0.1

REFERENCES

[1]Williams J T W, Pearce A, Smith A. A systematic review of fear of cancer recurrence related healthcare use and intervention cost-effectiveness[J].Psycho-Oncology, 2021, 30(8):1185-1195.

[2]Chelune, GJea. Self-disclosure and its relationship to marital intimacy. Journal of Clinical Psychology. 1984; 40(1):216–219. [PubMed: 6746933

[3] Prager, KJ. The Psychology of Intimacy. Guilford Press; New York: 1995

[4] Badr, H., Herbert, K., Chhabria, K., Sandulache, V. C., Chiao, E. Y., & Wagner, T. (2019). Self-management intervention for head and neck cancer couples: Results of a randomized pilot trial. *Cancer*, *125*(7), 1176-1184. https://doi.org/10.1002/cncr.31906

[5] Carlson, L. E., Rouleau, C. R., Speca, M., Robinson, J., & Bultz, B. D. (2017). Brief supportive-expressive group therapy for partners of men with early stage prostate cancer: lessons learned from a negative randomized controlled trial. *Support Care Cancer*, *25*(4), 1035-1041. https://doi.org/10.1007/s00520-016-3551-1

[6] Couper, J., Collins, A., Bloch, S., Street, A., Duchesne, G., Jones, T., Olver, J., & Love, A. (2015). Cognitive existential couple therapy (CECT) in men and partners facing localised prostate cancer: a randomised controlled trial. *BJU Int*, *115 Suppl 5*, 35-45. https://doi.org/10.1111/bju.12991

[7] Duan Suwei, Q. L., Zhang Aiping, & Dou Jingjing. (2022). Effects of binary coping intervention on patients and their spouses after breast cancer surgery. *Journal of Nursing*, *37*(04), 71-74.

[8] Nicolaisen, A., Hagedoorn, M., Hansen, D. G., Flyger, H. L., Christensen, R., Rottmann, N., Lunn, P. B., Terp, H., Soee, K., & Johansen, C. (2018). The effect of an attachment-oriented couple intervention for breast cancer patients and partners in the early treatment phase: A randomised controlled trial. *Psycho-Oncology*, *27*(3), 922-928. https://doi.org/10.1002/pon.4613

[9] Porter, L. S., Baucom, D. H., Keefe, F. J., & Patterson, E. S. (2012). Reactions to a partner-assisted emotional disclosure intervention: direct observation and self-report of patient and partner communication. *J Marital Fam Ther*, *38 Suppl 1*, 284-295. https://doi.org/10.1111/j.1752-0606.2011.00278.x

[10] Porter, L. S., Keefe, F. J., Baucom, D. H., Hurwitz, H., Moser, B., Patterson, E., & Kim, H. J. (2009). Partner-assisted emotional disclosure for patients with gastrointestinal cancer: results from a randomized controlled trial. *Cancer*, *115*(18 Suppl), 4326-4338. https://doi.org/10.1002/cncr.24578
